# The Action of Alcohol upon the Human System

**Published:** 1867-05

**Authors:** R. L. Walston

**Affiliations:** Paris, Ill.


### CHICAGO MEDICAL EXAMINER.

N. S. DAVIS, M.D., Editor.

VOL. VIII. MAY, 1867. NO. 5.

### Original Contributions.

ARTICLE XX.

### THE ACTION OF ALCOHOL UPON THE HUMAN SYSTEM.

By R. L. WALSTON, M.D., Paris, Ill.

Read before the Esculapian Society.

A few weeks ago, I observed that the National Medical Association made the announcement that, for three successive years, committees had been appointed to make a report upon the action of alcohol upon the human system, but that no report had ever been offered—that each appointment had proved a failure. In all our medical literature, there is a complete dearth upon the subject; there is but one monograph in our language that has even a *bearing* upon this topic. It is for these reasons that I now bring my feeble offering and lay it as a willing sacrifice upon the altar of our profession; not that I entertained for a moment the vain aspiration that I should be able to supply the desideratum, but that I might incite others to do so. It is a subject surrounded by difficulties of which I at first had no conception, and to have prepared a labored, elaborate address upon this subject would have required much thought, great research, and some sparklings of genius. Time for thought I have not had, research I have never made, and to genius I lay no claims. But should I succeed in throwing a single ray of light across your path, or inspiring a single emotion within your bosoms, to impel you forward in the investigation of this subject, my object is gained.

By alcohol, is meant the intoxicating principle of all spirituous or fermented liquors. It is an unnatural product, not found free in nature, but is always prepared at the cost of the destruction or decomposition of other substances. It has had an existence almost ever since the world was. We are informed in the *Book* that, away down in the dim twilight of the world's history, one Noah was *drunken* with *wine*, and, true to the instincts of such condition, he steeped his ingrate soul in that most unnatural crime. You may sweep back through channels of history into the dark aisles of tradition until superstition and ignorance become an impenetrable veil, illumined only by a few flashings from the Jewish theocracy or gleams from the Trojan fable, and you will find that this mysterious spirit has ever dwelt among the destinies of mankind, at one time sitting, like the hellish imps of discord, gibbering upon the hearthstone, and at another, like the angel of mercy, at the bed of the dying. From its multiple phases, moralists have been wont to represent it as Proteus, or the Old Man of the Sea; but if such figure be admissible, it is dependent upon the shape he assumes, whether he is the Goddess Hygea or the Juggernaut of destruction.

The history of nations gives evidence in behalf of these statements. Greece rolled the car of ambition to the very acme of anticipated greatness and glory, and beheld, with complacent smile, her mighty achievements and unequaled strength; but when she pressed to her lips the intoxicating goblet, her brain ran wild, and she, like an avalanche loosed from some Alpine peak, plunged into the yawning chasm below. Her glory has departed, and, from her mournful ruins, from her desolated rubbish, there comes forth a voice so potent as not to be mistaken. Alexander, the world conqueror, surrendered to the winecup, and died as the fool dieth; his generals divided the known world between them and deluged it with blood. War is the great work, or rather the sport and triumph of death—the terrible generalissimo of evil, and his aide-de-camp is wine. But never, in the world's history, did intemperance reach its climax of sin and violence, of shameless crime and misery, never did it spread over the earth so like an epidemic, until our new, fair, fresh continent yielded the product of its rustling cornfields to the distillery. The vigorous and virgin soil yielded profuse harvests of grain, and the enterprising Yankee manufactured that bounty, which heaven had given for food for man and beast, into alcohol. And thus we had a surfeit of this precious remedy, and hence its shameful and unfortunate abuses. We may imagine the wealthy old Dutchman, sitting under his ample porch, from which the sun's rays were all excluded by his teeming fruit trees, gazing out over his luxuriant fields of wheat, of rye, of corn, and of clover; his cattle slumber in his rich pastures, and his sheep whiten the hillsides. He soliloquizes:—"Oh, that I could find a market for my rural wealth! My barns are already filled, what shall become of my abundant stores?" His satanic majesty is always accommodating to the rich, and he inspires a foreign schoolmaster to come to his aid; and thus the devil and the Scotchman made the wine-presses to run and gush like rills and fountains with this poisonous beverage, which heaven had appointed as a blessed remedy to quicken the sluggish pulse, sustain the failing nerves, and unwrinkle the gathering brow of care.

It is a well known but mysterious fact, that certain agents, when taken into the system, have, first, an elective, local action —that they manifest an affinity, a special preference in favor of some organ or parts of an organ; for example, subnitrate bismuth acts directly and locally on the extremities of the sentient nerves of the stomach, veratrum on the heart, and belladonna manifests its preference in favor of the pupil of the eye, and alcohol is plainly shown to have an elective affinity in favor of the brain and nervous system. Why and wherefore this mysterious action, none may know. It is as mysterious as language, and all attempts to explain it is like the teachings of calculus, that zero above zero is the origin of quantity; it but makes darkness visible, and belongs, properly, to the ken of a future state.

The physical change wrought in a simple piece of animal tissue by the direct action of alcohol is that of corrugation, which change is due to the difference in the affinity which the tissues have for alcohol and water. The change takes place by endosmosis and exosmosis, the water passing out and the alcohol passing in in about two-thirds the quantity of water before contained; hence the corrugation or shrinking. The reason that the tissue absorbs less alcohol than it, in its normal state, contained water, is due to the coagulation of its albumen. This physical change makes an important alteration in the chemical relation of the different elements of the tissues, and since a certain definite proportion must exist between the constituents before nutrition can possibly take place, you see at once how alcohol in the tissues of the animal body must derange all the nutritive functions. The change in the albumen does not take place in the *vitalized* tissues only in the most extraordinary alcoholic potations; but that such changes do take place, in rare instances, none can doubt. No considerable physical change can take place without seriously deranging the vital function of the changed organ or organs. Thus it is, that dogs are killed by the injection of the smallest amount of alcohol into the veins by the clotting of the albumen. Albumen is not, in its own state, organizable, but is changed to fibrine and then to organized tissue, and anything that destroys or prevents the change of albumen to fibrine necessarily intercepts nutrition and cuts off all repair of tissue, and causes starvation and death of *nerve-tissue*, for we shall show, by-and-by, that these phenomena are manifest in the nervous system, as is distinctly seen in the web of a frog's foot, by the microscope, when the frog is intoxicated, by which we learn that alcohol, in a very dilute state, exalts, for a time, vital action, but is soon followed by a corresponding vital depression, from which the system is long in rallying, so that there is much less vital force generated in a given time with than without alcohol.

Some suppose that alcohol, in small and frequent doses, is a tonic; but of this we have no proof. The property of tonic remedies, is to increase the vital contractility of muscular tissues, but especially of the bloodvessels, and in this we have, at all times, a relaxation of the muscular coat of the bloodvessels and a dilated state of the vessel. If a piece of nerve-tissue be subjected to the action of galvanism till it loses its excitability, and then is dropped for a moment into strong alcohol, the alcohol seems to arouse the expiring vital forces, and it is again excitable by the galvanism; but if first acted upon by the alcohol till it refuses, it then falls into a state of hopeless depression, from which no galvanism can ever arouse it. Small earth-worms and puppies are as surely killed by being intoxicated as if the brain was parboiled in an alkali. The membranes of the nervous centres are so tender as to allow the escape of alcohol through them, and the albumen of the brain is thereby coagulated and we have death as a certain result. Alcohol has one peculiar effect upon the blood, which I wish here to mention, as explanatory of something yet to come. When alcohol is taken continuously, it not only coagulates, to some extent, the albumen, as before shown, but it so contracts the red globules as to lessen their capacity for containing the hematine, or coloring matter of the blood, and it is squeezed out into the liquor sanguinis, where it retains the carbonic acid and rejects the oxygen presented to it at the lungs, and hence we have that dusky, dark-blue, cyanotic condition of habitual drunkards.

Intoxication is manifestly, as the etymology of the term designates, a condition of poisoning, and may, for convenience, be divided into three stages. The first is characterized by an increased activity of the heart's action; full, rapid pulse; flushed face and heated surface; with a general exaltation of all the organic functions; but it is most obvious that the encephalic centres are in a state of exalted function, evinced by the loquacity, rapidity and variety of thought, exhilaration and hilarity, and animated countenance and gestures. It is in this state that the old adage, *in vino veritas*, is so clearly manifest in the prevailing inclinations and proclivities being made known. "The irritable and ill-tempered become quarrelsome, the weak and silly are boisterous with laughter and mirth;" they fancy themselves insulted, take fire within, rave, threaten, and then come to blows. Seneca spoke of them, as those who let in a thief at the mouth to steal away the brains—for the right hand is lifted against the dearest friend, perhaps the wife of his bosom, and in one rash hour performs a deed that haunts him to the grave. This stage is nearly allied to incipient phrenitis, or mania. This exaltation is not a sustained exaltation of nervous forces, but a perversion of them. If the stimulus be now pushed any farther, the second stage is ushered in, where delirium begins and all control over the thoughts is lost, and the man becomes confused and a little muddy, intellectually, (and sometimes physically,) with disordered reasoning powers and the victim of hallucinations, of vertigo, and double visions. Vomiting occurs here, from a poisonous condition of the brain-substance, and this is nature's effort to relieve herself; but *similia similibus curantur* gives him a little more alcohol. The third stage is a total cessation of sensorial power and action, a profound coma, and this may be of every degree, from the sound, deep sleep of nature, from which the patient may be aroused to partial consciousness, to that deep, stertorous breathing; sound, apoplectic coma; tetanic convulsions, and death. When a fatal case occurs, it is plainly one of apoplexy, or a breaking down of cerebral power by effusions; the countenance wears a disgusting, bloated, tumid, livid appearance; the eyes protruding, open, vacant, and staring; and the lips blue and everted; though, in some instances of alcoholic poisoning, it would seem that it was a result of the action of the poison on the nerves of the stomach, particularly those of the sympathetic system, thereby stopping the heart's action, the patient dying of syncope rather than asphyxia. This is a state of *depression*, not *oppression*, as in apoplexy, and, in this condition, he who *bleeds his patient kills him.* Alcohol, as already shown, has an elective affinity in favor of the brain and nervous system, as other articles have a preference in favor of other organs or parts of organs. Its preference is first in favor of the cerebrum, the seat of intellect and reason. This is clearly evinced in the first stages of intoxication—quickened mental action, rapidity of thought, volubility of speech, and, temporarily, an increased mental power. It selects the cerebrum, as strychnia does the spinal cord; but push it to the second stage, and it reaches, by sympathy, the sensory ganglia upon which the cerebrum is superposed; and in the third stage, the medulla oblongata is involved, as evinced by the stertorous breathing, full, slow pulse, and a general sluggish movement of all the vital organs.

The skin, the kidneys, and the lungs are now taxed to their utmost endurance, to eliminate this poison from the system; but, unfortunately, they succeed but partially, for the reason that the glands, the real scavengers of the system, refuse to take it up, unless it is the kidneys partially, and that is a disputed question; and small quantities of alcohol have been so intimately incorporated into the nerve-tissues as to pass out only by a change of nutrition. But alcohol being a ready combustible, attacks the oxygen from the blood and other tissues, and is thereby changed by combustion into carbonic acid and water, to which combustion the system is mainly due for its increased heat.

I shall now pass to notice, briefly, some of the diseases of the brain and mind, induced by the *abuses* of alcohol, for it is the cause of more such diseases than all other causes combined. The delirium ebriosum of DARUM I shall not notice, it being only an exaggeration of one of the ordinary forms of excitement in common intoxication, and will generally pass away in a day or two, if the wildness and ferocity of the patient be restrained by anodynes and gentleness. But there is a condition intervening upon a continued state of over-excitement of the nervous system, known by us all as delirium tremens, which is so named from the tremulousness and tremor of the muscles, which is always the result of exhaustion—the exhaustion of nervous fever. But delirium tremens may originate in two ways:—It may originate from a continued application of the stimulant till the battery of nervous power is exhausted and ceases to respond to the stimulant; or it may originate from a sudden withdrawal of the stimulant when the supply of nervous force is so nearly exhausted as to be unable to act without its wonted stimulant, and it is in the latter condition only when you may relieve the patient by goading the brain a little further, by giving him a little more whisky. But the cure is, to put the brain in splints, and keep it at perfect rest, till it can recuperate and generate natural forces enough to run this little piece of divine mechanism. This failure of nervous force is plainly manifest in every case of delirium tremens. There is little or no heat of the head or flushing of the face; the surface is cool and humid, and even chilly and cadaverous; the pulse is small, frequent, and weak; the temper is not violent, but there is anxiety and general apprehension of evil. Most physicians have had patients attempt descriptions of this condition. One, I well remember, which was a vivid picture of a fiendish group of phantastic horrors and chilling agonies, which no pen could describe, even were it guided by the hand of a Dante. If some terrible monster enters your chamber at an unseasonable hour and threatens you, if you hear its footsteps and feel its hot breath, you arm yourself and go into the conflict, and every stroke does you good; but if you approach and strike it, and your good broadsword goes down through it as through a wreath of smoke, or it evades your grasp like a flake of down, or, like Milton's angels in the battle of Heaven, which, though a thousand times cloven, is still uncleft, you recoil from such an unequal conflict and yield yourself to such a deathless conqueror. And such being the nature of this dreadful affection, we have no difficulty in understanding how the habitual use of alcoholic liquors becomes one of the most frequent causes of insanity or settled derangements of the mind. It acts, as the statistics of lunatic asylums all go to show, both as a predisposing and exciting cause, in a greater number of cases than all other causes combined. And this is due more to the production of a disordered state of the nutrition of the brain and nervous system, than in any other way. I might here go on and give the statistics culled from reports of the various asylums in the United States and Great Britain, but time will not permit. But by the law of hereditary tendency, or the transmission of condition, tendencies and appetites, it is remarkably manifest in the offspring of drunkards. Thus Plutarch says:—"one drunkard begets another;" and Aristotle says, that "drunken women bring forth children like unto themselves." Diogenes said to a stripling, somewhat crack-brained and half-witted:—"surely, young man, thy father begot thee when he was drunk." Shakespeare seemed to recognize the law of hereditary transmission of qualities so much insisted upon in the text:—"Come on ye cowards; ye were got in fear, though you were born in Rome." It is a matter of history, worthy our notice, that the child of an habitual drunkard was never known to attain to greatness.

That there is a certain condition of exhaustion of nervous forces, a starved condition of the nutritive power of the brain, which, when transmitted, results in idiocy and imbecility, is settled beyond a doubt, and is accounted for upon principles much better known to science than the law of congenital nevus, or the marks produced upon children in utero, by impressions made upon the mind of the gestating mother. Dr. HOWE, in his report to the legislature of Massachusetts, makes the following characteristic remarks:—"The parents of 300 of the idiots were ascertained, and 145 were reported as known to be drunkards. Such parents give a weak and lax mental constitution to their offspring, who are deficient in vital energy, and predisposed, by their very organization, to cravings for stimulants." This is my argument exactly, that having a low vital energy, a deficiency in real brain force, they have a natural thirst for intoxicating liquors, a longing for something to goad up the brain till it will yield the amount requisite for the daily demand.

If, now, we turn our attention to the inflammatory condition of the brain and its membranes, I presume every physician has a wide and melancholy field of illustration. Inflammation of the brain and its membranes is a usual concomitant of a protracted debauch; or, if the unfortunate recovers short of actual inflammation, he is like to suffer from a protracted state of passive congestion of the brain substance, producing that weight and heaviness of which we so often hear complained. But time does not permit me to dwell longer, and I must hasten on to touch upon a few prominent points in the diseases of the alimentary canal, produced by the too free and frequent imbibition of alcohol.

The effect of alcohol upon the healthy tissues of the stomach are more plainly manifest in the specimen I here show you, than I could illustrate in a whole evening's lecture. Here is the stomach of a drunkard, otherwise a healthy man. I took it from his body with my own hands. It is true, he suffered with all that train of diseases which accompanies such a life, but it was, emphatically, whisky and obesity of which he died and went to hades. The mucous coat is a perfect mesh of bloodvessels; it is thickened and indurated; the mucous coat is raised up, as it were, to protect the extremities of the gastric nerves from alcohol; there are ulcers and little abscesses filled with a hard granular pus, which has burrowed between the different coats, almost dissecting them from their normal adherence to each other. Such is the havoc made in the stomach by alcohol; and the effect upon the duodenum and other bowels is like unto this. But it is useless for me to continue to enumerate the different diseases of specific organs of the body, it will suffice to say, that it produces amyloid degeneration of the liver, cirrhosis, hydro-peritoneum, fatty liver, epilepsy, muscular tremors, gastritis, pyrosis, various dyspepsias, and the lesions of the kidneys known as "Bright's disease," and last, but not least, alcoholism *favors the production* of nearly all other diseases, by lessening the power of resisting their causes, and contributes to their fatality by impairing the ability to tolerate and overcome them.

Drunkards readily succumb to inflammatory diseases of every variety. Surgeons are always desirous of getting union by first intention, which union they never get in habitual drunkards, on account of the impaired plasticity of the blood. But some are, doubtless, ready to ask the question:—Has alcohol no virtues as a remedial agent? I answer, yes, it is indeed a saver of life unto life: but it is then a benign agent, as a poison, as a medicine, and all medicines are poisons. There are a thousand instances and conditions wherein this powerful arterial stimulant may be used in restoring the injured or wounded from severe shocks, or sustaining the failing life force, or fanning the expiring flame of life into an increased fervency and brilliancy. In chronic diseases, in low fevers, and in extensive superficial burns where, by the direct action of fire, the temperature falls to a degree below the point consistent with vitaltality, a little stimulant may be administered with magic results in sustaining the heart's action and nerve forces till reäction is manifest. But such administration should always be under the direction of an intelligent physician.

There formerly existed some doubts whether the human system could not keep up a sustained effort of its physical and intellectual powers by the aid of alcoholic stimulants longer with than without them, whether or not it did not increase the aggregate of man's endurance. But however many doubts might have lingered in the public mind formerly, it seems that the history of the last five years would have dispelled them all. Our soldiers were not goaded on to victory by any such degrading impulses. They underwent exposures and privations, endured hardships and fatigues, made wearisome marches beneath the burning rays of a tropical sun, exposed to the diseases of a high latitude, all without a murmur. They faced enemies, fought battles, and achieved victories, the like of which the world has never known—they carried bibles, not bottles—a sound conviction of a noble duty was their only stimulant. But does not alcohol increase man's endurance in a continued mental effort? With most persons it produces a temporary exaltation of mental activity, lighting up the scintillations of genius into a more brilliant flame, or assisting the prolongation of a *single* mental effort when the powers would otherwise be exhausted; but it must be remembered that it it is not in any brilliant corruscations or unnatural irradiations of wit or humor in which the merit lies, but it is in the continued fervor of the glow which drops the rays of light into the dark corners of the world's great heart, lighting it up with the lamps of wisdom, and sowing seeds which bring forth the golden harvest. If spirits would make poets and orators, the world would be full of them. The lights of genius may be untimely burnt out and forever extinguished, as in the case of Mozart, and Haller, and Byron, and Poe. These men gave manifestations of music and poesy in spite of, and not on account of, alcohol. Call Homer from the dead, interrogate that blind old bard of Scio's rocky aisle, as he rolled his quenched eyeballs to find a ray of light, and ask him if that mellifluous epic which he warbled through the listening cities of his native seas, was the fruit of alcoholic potations; and then Milton, a second Homer, but a greater than the first, was it alcohol or celestial lights which shone inward and raised up things invisible to to mortal sight. Do the works of Locke and Bacon bear the impress of a heated brain? Are they volcanic productions? No sir, the Novum Organum and the Essay on the Understanding, give evidence of minds purified by a refiner's fire.

The brain is the organ of the mind, and whatever interrupts the nutritive functions of that organ or changes its sensitive, delicate textures, prevents the mind's ready response to moral ideas and lessens the power and correctness of all intellectual operations. Thought is the true standard of intellectual excellence. 'Tis thought that has said to the wilds of America, blossom, and almost instantly it has become as the garden of Eden. It says to the mountains, be ye opened, and instantly its bowels of rock are blasted out. 'Tis thought that outstrips the wing of lightning, and speaks like the voice of God. It defies volcanoes and storms, and laughs and makes out warrants and executions in its burning path; it tameth the tiger, and maketh a plaything of the lion. 'Tis this, of all things, which most challenges our admiration. We look at the idiot, or even the temporarily insane, and feel that it is a terrible thing to have the reason extinguished, or that some crushing power should rest upon the intellect, paralyzing it. It would be no compensation for us to know that our children would grow up with all the beauty of an Antinous or the giant proportions of an Apollo, did we not expect intelligence to accompany their maturity. Even the club-footed and deformed are loved and courted, if but this God-like quality is left them—the power to think.

I, for one, believe in an aristocracy, social castes, and an hereditary nobility. But these distinctions should not be erected upon the crumbling basis of wealth. It should be an aristocracy of intellect, a caste of thought, a nobility of mind, and it is plainly the duty of physicians, as champions of moral ideas and devotees of science, to enlighten the people upon the action of every agent which tends to corrupt the fountains of virtue in themselves, or to fetter and trammel the intellect of their offspring. And we have but to cast around us, to see that alcohol, more than all other influences, tends to corrupt and pollute the purest avenues of society, rendering those minds vulgar and plebeian which otherwise would be dignified and noble. "This most excellent canopy, the air, this brave, o'er-hanging firmament, this majestic roof fretted with olden fire! What a piece of work is man: how noble in reason; how infinite in faculties—in form and moving, how express and admirable; in action, how like an angel; in apprehension, how like a god; the beauty of the world, the paragon of animals." We delve deep in the mysterious organic structure, but the glittering scalpel throws back the reflection, "there is a God, who made all this." But the correction of moral evils belongs not to science; and leaving the subjects of death—*the grim ferryman*—and man's accountability to God to the man of ethics, I shall offer but a single reflection more.

I desire not to take the *role* of Mentor, nor wear the Nessus' shirt of self-conceit; but I would be understood. What are we to do? We cannot ignore the use of this valuable agent of the text; in such attempt we but fly from Scylla to be wrecked on Charybdis. But guided by the light-house of science, and steering our bark in the middle of the channel of truth, we may use it with caution, at all times exercising a wise discretion as to when and how it shall be administered, if not, we will increase human sufferings, while our mission is to ameliorate them. The physician goes by the ditches and gutters of pollution, but not like the arrogant priest and haughty Levite, who did not so "much as look that way," or walked on the other side, but like the good Samaritan, he may lift mankind from the trenches of vice as well as the touch of pain and disease. He knows their foibles and failings, and if he is a *physician* and treats the whole man, he addresses his remedies to the failing part, be that attribute or organ. But alcohol has an action directly upon our profession, one of which I have not spoken. There is an aspersion which comes against us from the great heart of the people, which is more loathsome than the sores of Lazarus, or the leprosy of Naaman—one which can neither be hid in "Abraham's bosom," nor purged away in the pool of Siloam—it is this, that the profession, as a profession, is under the ban of intemperance; that the footsteps of the drunkard are wont to be heard in the chambers of woe. Ah! be it said, to our everlasting shame, that the pestiferous breath of alcoholic potations is exhaled around the bed of the dying. Will we not, as a profession, arise and shake this stain from our garments, as the lion shaketh the poisonous vapors from his mane, and hurl back the foul aspersion into the faces of those who make it. Let us be willing to labor for the advancement of the profession—retarded by individuals, it may be, but disgraced it shall never be.

